# Retraction: The role of Janus kinase/STAT3 pathway in hematologic malignancies with an emphasis on epigenetics

**DOI:** 10.3389/fgene.2023.1285098

**Published:** 2023-09-04

**Authors:** Frontiers Editorial Office

The journal retracts the 2021 article cited above.

Following publication, concerns were raised regarding the contributions of the authors of the article. Our investigation, conducted in accordance with Frontiers policies, confirmed a serious breach of our authorship policies and of publication ethics; the article is therefore retracted.

This retraction was approved by the Chief Editors of Frontiers in Genetics and the Chief Executive Editor of Frontiers. The authors do not agree to this retraction.

